# Supplementary figures and images for: Centrality of Hygienic Honey Bee Workers in Colony Social Networks
Source: Insects. 2025 Jan 10;16(1):58. doi: 10.3390/insects16010058 (PMC11766216; doi:10.3390/insects16010058)

## Supplementary Materials

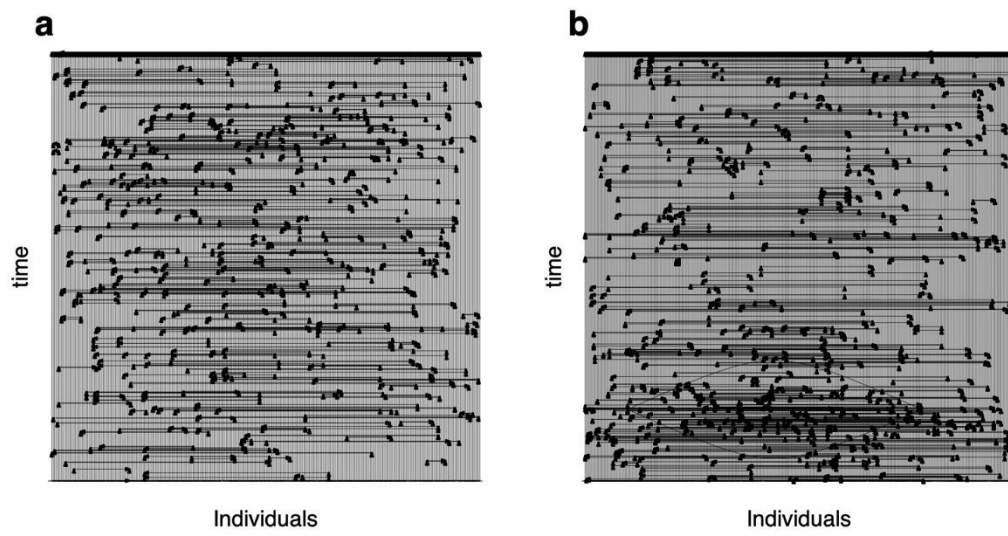

**Figure S1.** Time-ordered social networks from colonies 1 (a) and 2 (b).

Supplement: Supplementary file 1 [file insects-16-00058-s001.zip › insects-3357430-supplementary.pdf]
